# Supplementary material for: Needs of Alzheimer’s Charges’ Caregivers in Poland in the Covid-19 Pandemic—An Observational Study
Source: Int J Environ Res Public Health. 2021 Apr 23;18(9):4493. doi: 10.3390/ijerph18094493 (PMC8122957; doi:10.3390/ijerph18094493)
Supplement: Supplementary file 1 [file ijerph-18-04493-s001.zip › ijerph-1180459-SI.pdf]

Title: Potrzeby opiekunów osób cierpiących na chorobę Alzheimera w dobie epidemii Covid-19.

[Needs of Alzheimer's charges' Caregivers in Poland in the Covid-19 Pandemic]

Introduction:

The coronavirus epidemic has taken everyone by surprise. It has drastically changed the reality in which we live. We cannot fail to notice that the elderly and those with chronic illnesses are at risk. In the current situation, we must not forget the thousands of caregivers of people with Alzheimer's disease (AD-Alzheimer's disease) living in Poland who face a new reality and new problems.

I am a therapist who has been working with people affected by dementia for years. I cooperate with Alzheimer's Foundation of Lower Silesia in social projects and with nursing homes in Wroclaw. As a PhD student at the Faculty of Physiotherapy of the University of Physical Education in Wrocław, I try to transfer my knowledge into actions "here and now".

The questionnaire is fully anonymous. It consists of two sections. The first concerns your situation and information related to the epidemic. The second section is the PSS-10 stress questionnaire. Participation in the survey is voluntary.

The questionnaire takes no more than 10 minutes to complete. It does not require you to share personal data, medical records, sensitive data or re-contact. We rely on your observations and feelings about your new situation. Please answer honestly. Thank you for your support!

The purpose of this survey is to identify the needs of carers of people with AD that have arisen with the new realities. It is also a first step towards providing real support, both physically, mentally and socially, for carers and their clients

Without your help, it will not be possible to identify the real needs and challenges you currently face. This survey will provide a basis for action in the near future to improve the situation of caregivers and charges with Alzheimer's disease.

Section I

1. Which gender do you identify with?

- Female
- Male

2. How old are you? (insert number of years)

3. How old is your charge? (insert number of years)

4. In which form of family life do you currently function?

- Single
- Husband/wife
- Widow / Widower
- Divorced
- Separation
- Partnership
- Other / add options

5. What is your relationship to the mentee?

- Spouse

- Daughter/son
- Daughter-in-law/ Son-in-law
- Granddaughter/grandson
- Sister/brother
- Other/ add options

6. Where do you currently live?

- Large city (over 300,000 inhabitants)
- Medium size city (91 - 300 thousand inhabitants)
- Small town (up to 90,000 inhabitants)
- Village
- Other/add options

7. Did you live before the epidemic:

- Together with the chargee
- Together with the charge and other family members
- In another place than the charge

8. Are you currently living:

- Together with the chargee
- Together with the charge and other family members
- In another place than the charge

9. How many years has the charge had Alzheimer's disease or other dementia? (insert number of years)

10. How many years has the charge been in your care? (insert number)

11. What was the condition of the client before the epidemic?

- Mild cognitive impairment (relatively independent)
- Moderate cognitive impairment (requires assistance in daily functioning)
- Severe cognitive impairment (totally dependent on caregiver)

12. Did or did not the client's health deteriorate during the COVID-19 outbreak?

- Definitely deteriorated
- Rather deteriorated
- Hard to say
- Rather not deteriorated
- Definitely not deteriorated

13. Has or has not your health deteriorated during the COVID-19 outbreak?

- Definitely deteriorated
- Rather deteriorated
- Hard to say
- Rather not deteriorated
- Definitely not deteriorated

14. To what extent has the Covid-19 outbreak changed your daily life?

- Definitely changed
- Rather changed

- Hard to say
- Rather not changed
- Definitely not changed

15. have new needs emerged during the epidemic in relation to caring for someone with Alzheimer's disease?

- Yes
- No

16. In which area have there been problems in your functioning in relation to COVID-19? (please tick up to 3 most important answers)

- Protective measures (use of disinfectant fluids, wearing a mask and gloves)
- Health care (difficulties in visiting the doctor, buying medicines at the pharmacy)
- Caring for a family member in care related to the closure of care facilities
- Loss of job or change of job to remote work connected with care of person in care
- Daily errands (shopping, paying bills, cleaning)
- Difficulties in finding additional care for the person in care
- Difficulties in obtaining psychological assistance / social support
- No new difficulties have emerged

17. Have you received an offer of assistance in relation to the Covid-19 outbreak from any of the areas listed?

- Organisational help (doing shopping, helping with care, doing housework, etc.)
- Psychological assistance (telephone consultation, conversation, support group, etc.)
- Social support (social programmes, financial assistance, social support e.g. phone calls from friends, offering help)
- We did not receive any offers of help in connection with Covid-19
- Other/ add options

18. In the COVID-19 situation, have your concerns about the health of the charge increased or not increased?

- Definitely increased
- Rather increased
- Hard to say
- Rather not increased
- Definitely not increased

19. In a COVID-19 situation, have your concerns about your health increased or not increased?

- Definitely increased
- Rather increased
- Hard to say
- Rather not increased
- Definitely not increased

20. what kind of help do you expect in connection with the pandemic? (please tick up to 3 most important answers)

- Provision of financial assistance during the Covid-19
- Provision of psychological support for the carer during the Covid-1
- Provision of care for the carer during working hours
- Providing the possibility to stay in hospital with the patient with AD suffering from Covid-19
- Provision of care for the patient with AD if the carer becomes ill with Covid-19

- Introduction of educational programmes for carers related to functioning during the Covid-19
- Provide legal assistance to the caregiver
